# Hydrogen-induced plasticity in nanoporous palladium

**DOI:** 10.3762/bjnano.9.280

**Published:** 2018-12-10

**Authors:** Markus Gößler, Eva-Maria Steyskal, Markus Stütz, Norbert Enzinger, Roland Würschum

**Affiliations:** 1 Institute of Materials Physics, Graz University of Technology, Petersgasse 16, A-8010 Graz, Austria; 2 Institute of Materials Science, Joining and Forming, Graz University of Technology, Kopernikusgasse 24/I, A-8010 Graz, Austria

**Keywords:** electrochemistry, hydride formation, in situ dilatometry, internal-stress plasticity, nanoporous palladium

## Abstract

The mechanical strain response of nanoporous palladium (npPd) upon electrochemical hydrogenation using an in situ dilatometric technique is investigated. NpPd with an average ligament diameter of approximately 20 nm is produced via electrochemical dealloying. A hydrogen-induced phase transition from PdH_β_ to PdH_α_ is found to enable internal-stress plasticity (or transformation-mismatch plasticity) in nanoporous palladium, which leads to exceptionally high strains without fracture as a result of external forces. The high surface stress in the nanoporous structure in combination with the internal-stress plasticity mechanism leads to a peculiar strain response upon hydrogen sorption and desorption. Critical potentials for the formation of PdH_α_ and PdH_β_ in npPd are determined. The theoretical concepts to assess the plastic strain response of nanoporous samples are elucidated, taking into account characteristics of structure and deformation mechanism.

## Introduction

Material properties on the nanoscale can differ substantially from their bulk counterparts considering the increasing importance of surface effects. The high surface-to-volume ratios in such materials allow for the control of bulk features by surface modifications. Electrostatic charging or electrochemical (surface) reactions are possible ways to influence metal surfaces in a well-defined manner. Therefore, open nanoporous network structures are particularly suitable for property-tuning experiments in an electrochemical environment, due to a large contact area with the electrolyte and macroscopic sample dimensions. In nanoporous metals, the electrochemical control of actuation [1–3], resistance [4–6], magnetic moment [5,7], optical transmission [8] and selective chemical transport [9] have been reported in recent years, apart from the mechanical properties described below.

Dealloying, a selective dissolution process, has become an established technique to produce metallic nanoporous structures. By exposing a (binary) alloy to an etching agent, the less noble component is gradually removed, while enhancing the surface diffusivity of the other. Surface diffusion is of special importance for the formation of nanoporous networks, as passivation of the alloy surface due to a flawless coverage by the noble component prevents the etching process. After Erlebacher et al. delivered a full description of the dealloying process on an atomistic scale [10], the process itself has been extensively studied [11–13], while being used to prepare a broad variety of different nanoporous metals [14–15].

Lately, dealloyed nanoporous palladium (npPd) structures [16] have received attention in the literature as electrochemical actuator materials [17–20]. Due to the ability of palladium to host hydrogen atoms in its crystal lattice, such actuators show exceptionally strong, reversible expansion. This distinguishes palladium from other metals in the same group of the periodic table, such as platinum or nickel, which are only capable of adsorbing hydrogen mainly on their surfaces. In the literature, a solubility of hydrogen in palladium up to concentrations of about 0.7 (H/Pd) has been reported for loading from the gas phase at a hydrogen pressure of the order of 30 kPa [21] at 20 °C. In this compositional range two different phases coexist: At hydrogen concentrations below 0.02 (H/Pd) only the α-phase (solid solution, PdH_α_) is observed, while at higher concentrations the β-phase (hydride, PdH_β_) starts to form. At concentrations above 0.6 (H/Pd) only PdH_β_ is present. These critical concentrations were reported for bulk palladium samples at room temperature and may vary for nanoscaled or stressed systems [22]. The lattice constant increases during hydrogen sorption from 3.887 Å for pure palladium to 3.895 Å for PdH_α_ (maximum hydrogen concentration at 25 °C) and amounts to 4.025 Å in PdH_β_ (minimum hydrogen concentration at 25 °C) [21]. This significant increase in lattice constants, and thus volumetric expansion upon hydrogen uptake, makes palladium also an interesting element for hydrogen-sensing applications [23].

The mechanical properties of nanoporous samples have been extensively studied in the literature, especially for the model system of nanoporous gold (npAu). Reports on potential-controlled creep [24], fracture [25] and strength [26] in npAu add to the list of above-mentioned tunable properties in nanoporous metals. The deformation mechanism in such structures has been discussed in detail. Since nanoporous materials exhibit high surface-to-volume ratios, moving dislocations may escape crystals via the surface, which may lead to a scenario of dislocation starvation [27]. This dislocation starvation also implies that the work hardening mechanism, which is based on dislocation interactions, is not active. However, current literature suggests that the active deformation mechanism in npAu is dislocation slip [28] and that dislocation starvation is not effective at low strains in npAu. Despite the local dislocation activity in the ligaments, macroscopic plasticity, involving dislocations travelling larger distances in the network structure, is hard to achieve in nanoporous metals [15]. Plastic deformation in npPd has not been the subject of experimental studies up to this point. This work focuses on the strain response of npPd upon hydrogenation and aims to shed light on the active deformation mechanisms.

## Results

### Electrochemical characterisation

**Figure 1:**
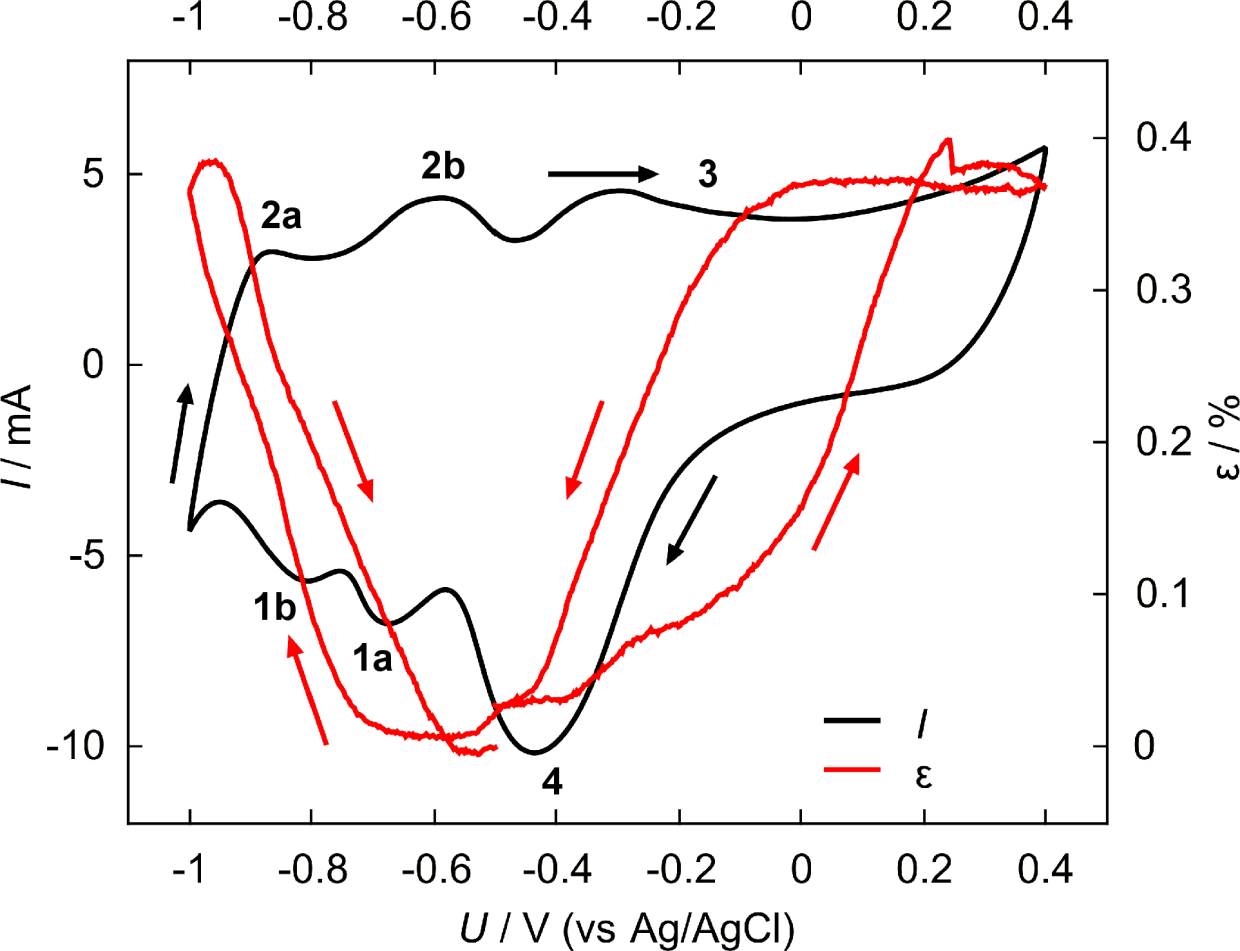
Current *I* (black) and corresponding strain ε (red) as functions of the applied potential *U* during a single voltammetric cycle for nanoporous palladium recorded at a scan rate of 0.5 mV·s^−1^ in 1 M KOH, zero on the strain axis was chosen in the double-layer regime, arrows indicate the cycling direction of the curves, numbered peaks are discussed in the text.

A typical strain response of npPd was measured using an in situ dilatometer setup during a cyclic voltammogram (CV) in 1 M KOH (see Figure 1). A CV probes the current as a function of the potential, which is varied up and down triangularly at a certain scan rate, which represents the slope of this potential variation.

### Potentiostatic hydrogen sorption

**Figure 2:**
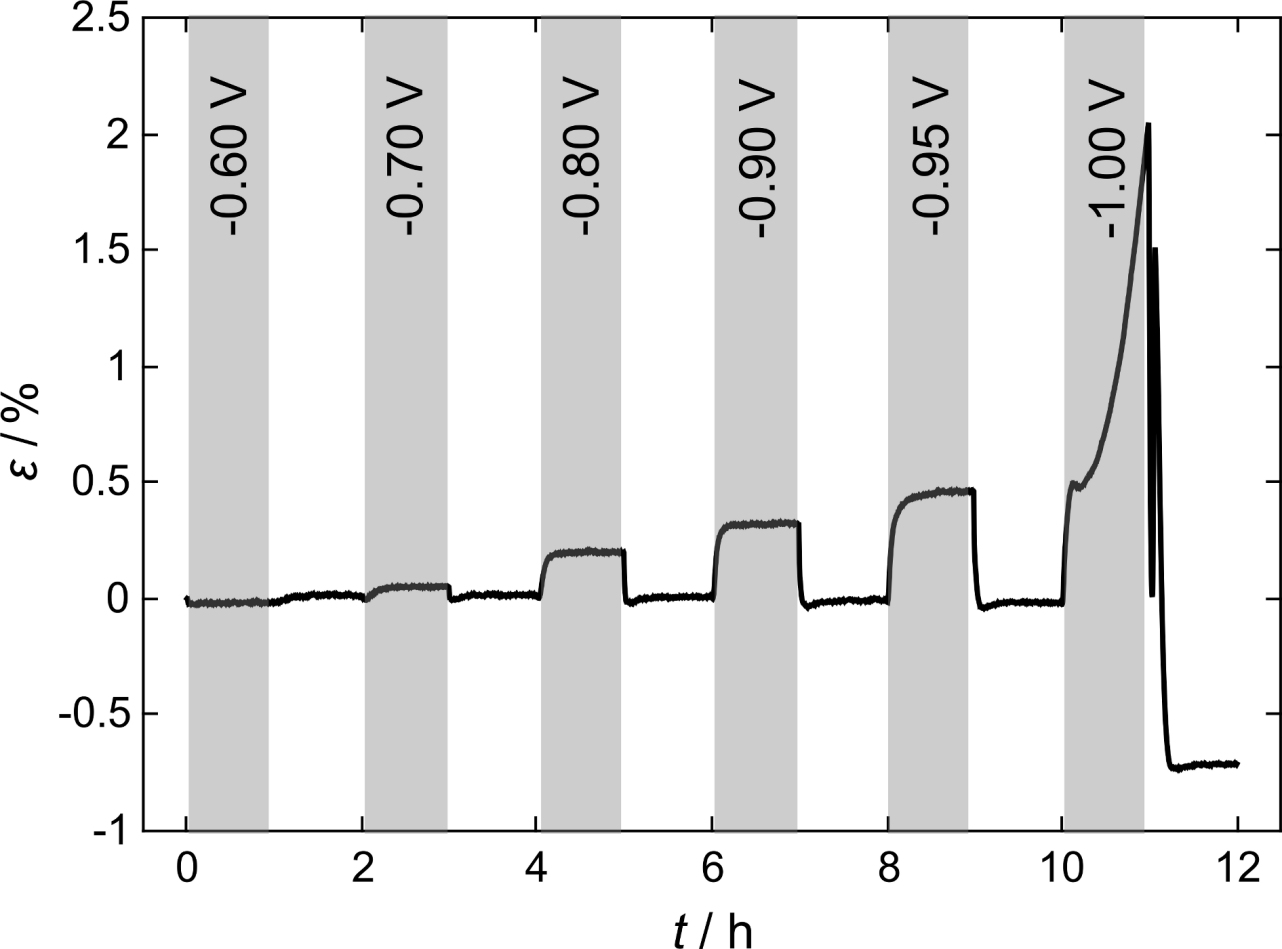
Strain ε as a function of the time *t* for a series of consecutive hydrogen absorption/desorption processes at constant potentials in 1 M KOH for nanoporous palladium. Loading processes are shaded grey, polarisation potentials are given in the grey bars, the unloading potential was fixed at −0.4 V.

The CV (black) in Figure 1 is typical for palladium and has been discussed in detail in prior studies [18]. Summarising briefly, the negative current peaks below −0.6 V (peaks 1a and 1b) correspond to hydrogen ad- and absorption, while the positive current peaks (peaks 2a and 2b) are attributed to the reverse process of hydrogen desorption. Above −0.4 V the broad flank at positive currents (peak 3) and the large peak at negative currents (peak 4) are features attributed to the adsorption and desorption of oxygen species. It should be noted that the comparably broad peaks strongly depend on the scan rate and exhibit a more distinct shape at lower scan rates. The strain signal (red curve in Figure 1) shows a reversible expansion upon hydrogen ad- and absorption below −0.6 V, while a quasi-reversible expansion, attributed to the formation of a palladium-oxide species on the surface, is observed at potentials higher than −0.4 V. The term quasi-reversible refers to the small offset accumulated after an oxide half-cycle, which can be attributed to weak anodic palladium (oxide) dissolution [29]. The length changes upon voltammetric cycling are in agreement with recent results for dealloyed nanoporous palladium from different base alloys [17–18]. The highly reversible strain response upon hydrogen sorption and desorption in the CV motivated a more detailed investigation in this potential regime. Figure 2 shows the strain measured during a series of hydrogen sorption (grey background) and desorption (white background) experiments with decreasing polarisation potentials, but a fixed unloading potential (−0.4 V). The potential for each sorption step shown is given in the plot. At a potential of −0.8 V a first significant reversible change in sample length of about 0.2% is observed, which increases with more negative sorption potentials to a value of about 0.4% at −0.95 V. Obviously, the last loading step at a potential of −1 V and the corresponding unloading procedure clearly differ in shape and height from the preceding ones.

**Figure 3:**
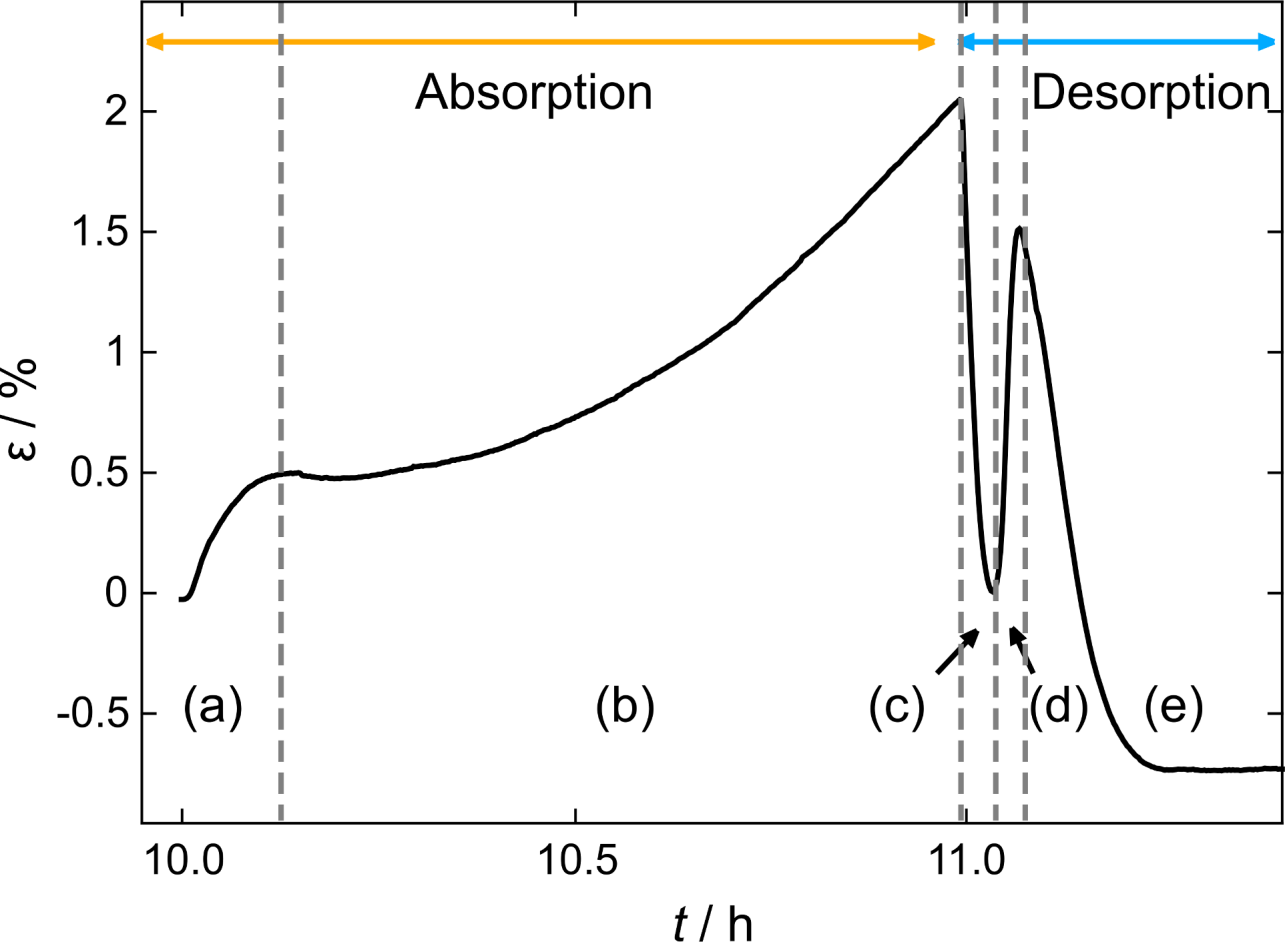
Enlarged view of Figure 2: Strain ε as a function of time *t* for electrochemical hydrogen absorption in nanoporous palladium at an anodic potential of −1.0 V followed by desorption at −0.4 V in 1 M KOH. Labelled regions are discussed in the text (regions (d) and (e): strain overshoot).

Figure 3 shows a detailed view of this hydrogen loading cycle at a potential of −1.0 V and the corresponding unloading procedure at −0.4 V. Upon hydrogen absorption two different processes can be distinguished in the strain curve: a steep increase up to a strain of about 0.5%, where the curve levels out (region (a)), and a parabolic increase from this point up to the onset of hydrogen desorption (region (b)). The beginning of desorption is marked by a sharp decrease in length (region (c)), followed by a short interval of sharp increase (region (d)), which stands in contrast to a simple picture where a monotonous shrinkage due to hydrogen extraction would be expected. The last stage of hydrogen desorption (region (e)) exhibits a similar slope to region (c) until the strain curve flattens out after complete desorption. The peak in the strain curve separating regions (d) and (e) will be referred to as strain “overshoot” in the following. An irreversible decrease of the sample length of about 0.7% was detected at the end of the experiment. This irreversible contribution to the total strain could be reproduced in measurements on further samples upon similar treatment.

### Critical potentials of palladium hydride formation

**Figure 4:**
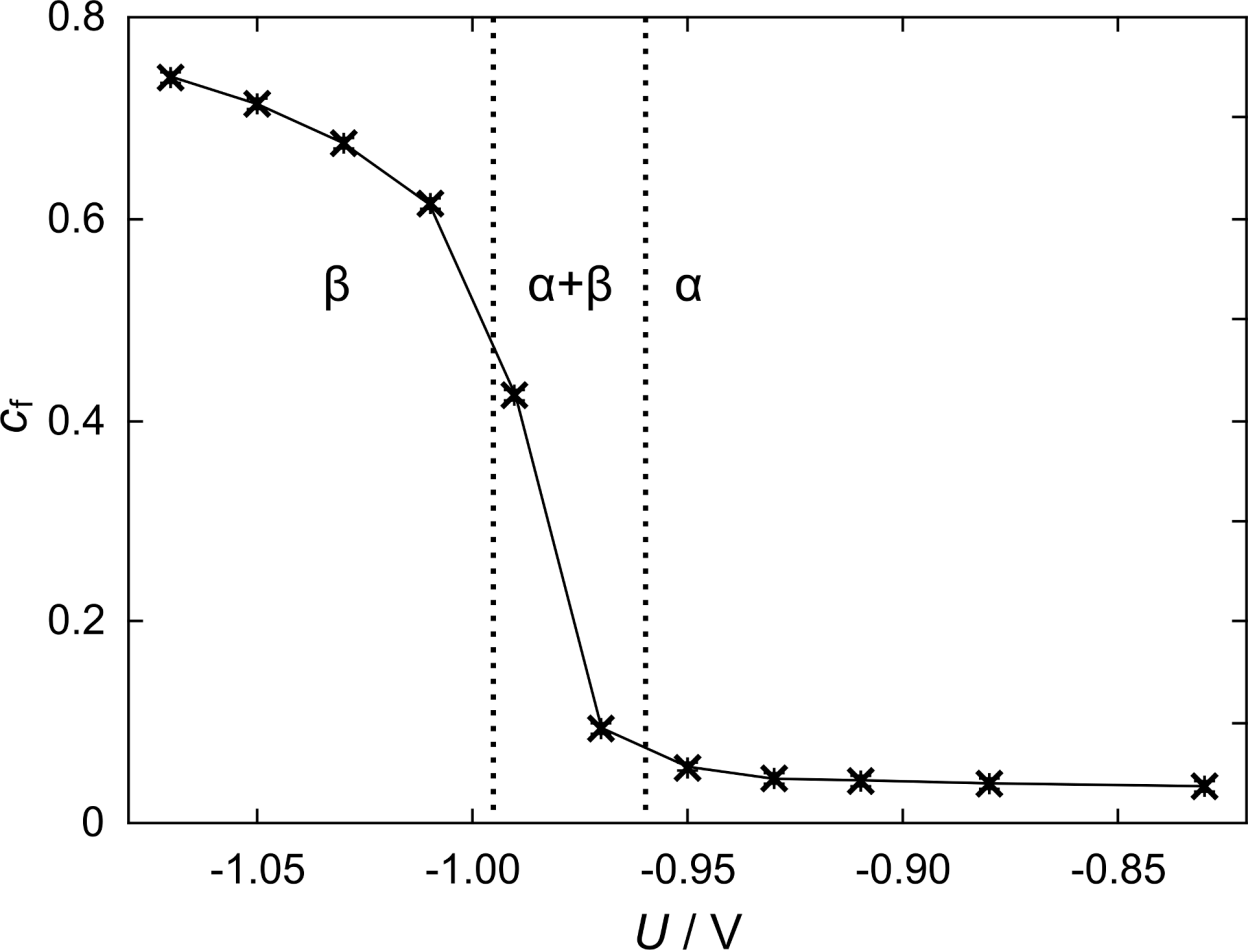
Atomic ratio of hydrogen and palladium *c*_f_ (H:Pd) plotted as function of the polarisation potential *U* for nanoporous palladium in 1 M KOH, dotted vertical lines separate the regimes of PdH_α_, PdH_β_ and phase coexistence.

A series of potentiostatic hydrogen loading and unloading measurements with decreasing loading potentials was carried out ex situ in order to distinguish the phases present in npPd at a certain potential. This procedure is thermodynamically equivalent to the measurement of pressure–composition isotherms (PCIs) upon hydrogen pressurisation. Since the parasitic process of hydrogen evolution contributes to the charge transfer during hydrogen loading, especially at strongly negative potentials, a calculation based on the recorded charge flow during hydrogenation might overestimate the hydrogen concentration in the samples. Therefore, the atomic ratio of hydrogen and palladium H/Pd (*c*_f_) was determined based on the potentiostatic desorption half-cycles. For each desorption step the imposed charge *Q* was determined by means of trapezoidal integration and converted into *c*_f_ considering the sample mass *m* using the following relation:

[1] $$c_{\text{f}}=\frac{Q\cdot M_{\text{Pd}}}{e\cdot N_{\text{A}}\cdot m}\;,$$

where *M*_Pd_ is the molar mass of palladium, *M*_Pd_ = 106.42 g/mol, and *e* and *N*_A_ are the elementary charge and the Avogadro constant, respectively. The hydrogen concentration is plotted as a function of the polarisation potential for npPd in Figure 4. Error bars, determined via the uncertainty of trapezoidal integration, are added in the graph, but are small compared to the plot symbols.

From the literature it is known that a distinction of palladium hydride phases is possible utilising potential–concentration plots as in Figure 4 [30–32]. In our nanoporous samples the PdH_β_-phase begins to form at potentials below −0.96 V. Pure PdH_α_ is present at potentials higher than −0.96 V, while PdH_α_ disappears at potentials below −0.99 V. Nanostructured materials can exhibit a significant narrowing of the miscibility gap in the phase diagram compared to the bulk metal [33], which can drive the onset of β-phase formation to higher hydrogen concentrations and/or lower potentials. For nanoporous palladium produced in this work, a *c*_f_ value of about 0.07 for the β-phase onset can be estimated from the data presented in Figure 4, which is in good agreement with a value of 0.06 determined for palladium nanoparticles [30]. The saturation of PdH_β_ formation and the corresponding depletion of PdH_α_ at a value below −0.99 V is consistent with values reported for palladium thin films [31–32]. The threshold values for the palladium hydride β-phase formation (−0.96 V) and the potential of α-phase depletion (−0.99 V) can be used for a phase distinction in current-controlled experiments.

### Galvanostatic hydrogen sorption

**Figure 5:**
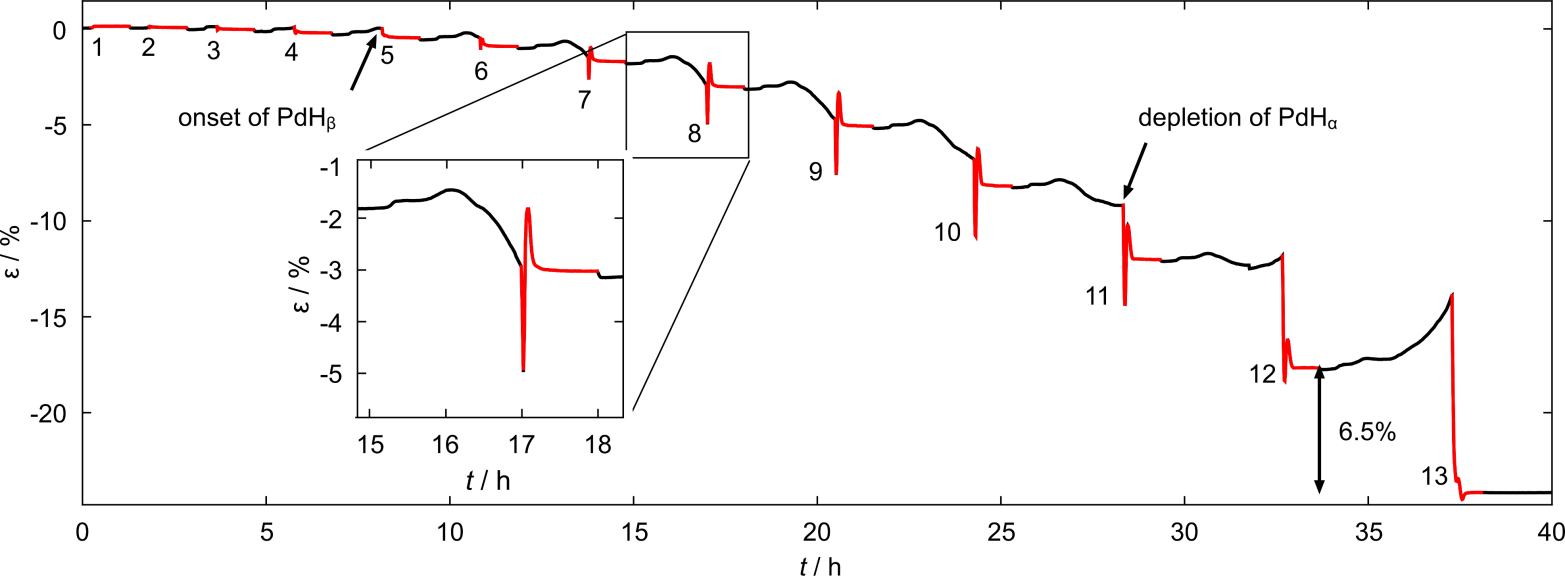
Strain ε as a function of the time *t* for a series of consecutive hydrogen absorption processes at a constant current of 1 mA and desorption processes at −0.4 V in 1 M KOH for nanoporous palladium. Desorption processes are marked red, the amount of charge during hydrogen loading (black) was controlled via the sorption time and increased in steps of 1 A·s from 1 A·s to 13 A·s.

In order to clarify the origin of the unusual strain overshoot observed in Figure 3, a charge-controlled sorption experiment was performed (Figure 5). A constant current of 1 mA was used to charge nanoporous samples to different total amounts of charge (from 1 A·s to 13 A·s in total, black curves) and, thus, hydrogen concentrations (compare Equation 1), while for desorption (red curves) the potential was held constant at −0.4 V similar to the potentiostatic experiment above (Figure 2). Monitoring the potential during the charging procedure revealed a transgression of the PdH_β_ formation threshold in the fifth loading cycle, which is marked as “onset of PdH_β_” in Figure 5. The potential of PdH_α_ depletion was crossed during the 11th loading cycle, which is also indicated in Figure 5.

Both strain features present in potentiostatic experiments during desorption, namely the strain overshoot and the irreversible strain offset after a loading/unloading cycle, can be followed in Figure 5 evolving over time. During the first four cycles the strain amplitude upon absorption remains small of the order of 0.2%, with no obvious irreversible contribution. Furthermore, no overshoot can be detected in these initial cycles. In the fifth cycle, the strain upon absorption increases up to 0.4% and an irreversible strain contribution after desorption of about −0.3% emerges. Starting with the sixth cycle the strain overshoot becomes more and more pronounced each step (red curves in Figure 5). Moreover, the strain offset grows drastically up to a value of 6.5% for the last desorption process as marked in Figure 5. An additional feature that has not been observed in the potential-controlled experiment (compare Figure 2) is the sample contraction upon hydrogen absorption in the galvanostatic experiment (black in Figure 5, enlarged in the inset). Figure 5 suggests that the presence of the PdH_β_-phase might have a critical influence on the irreversible length changes in npPd, which will be analysed in the Discussion section.

**Figure 6:**
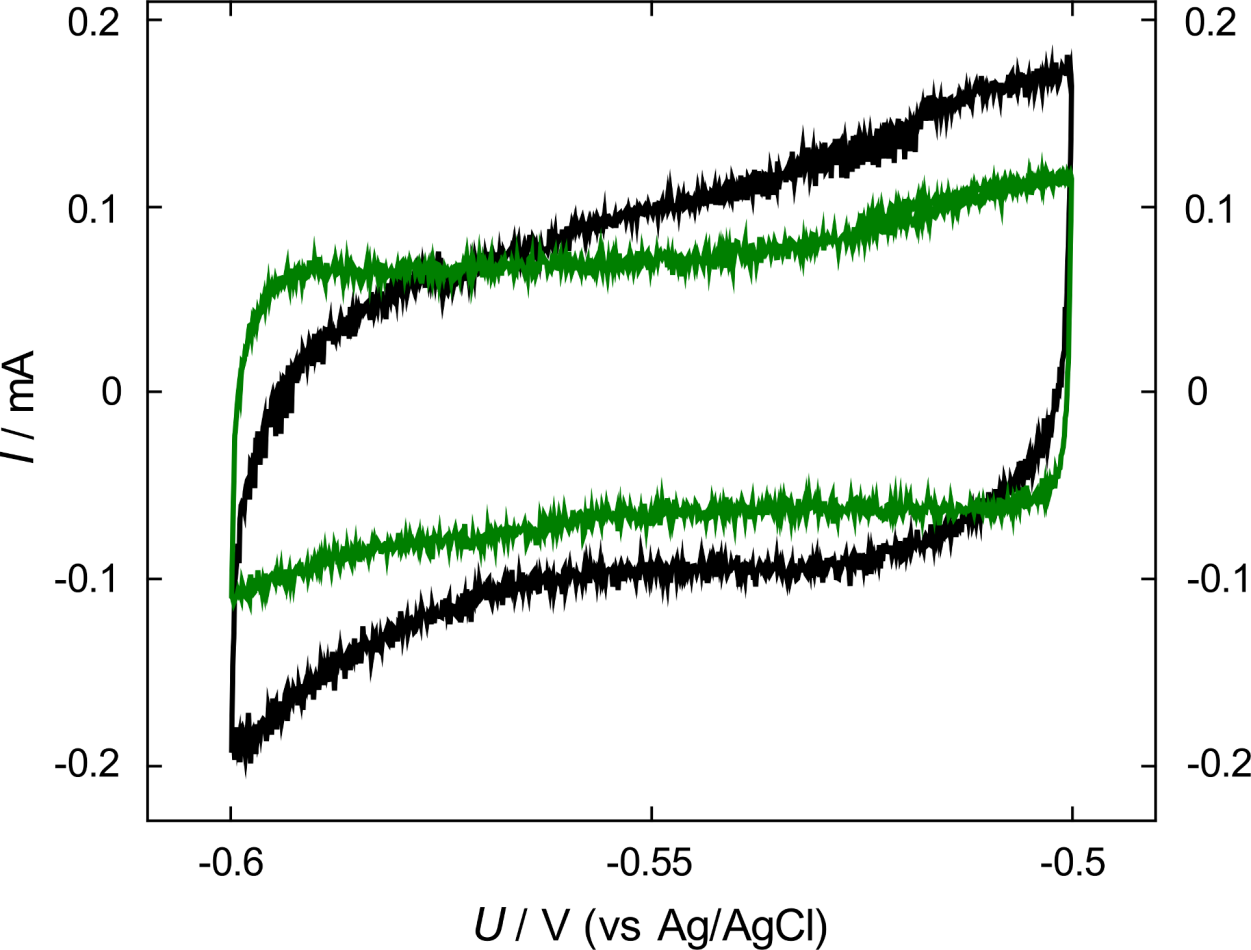
Current *I* as a function of the applied potential *U* during a single voltammetric cycle for nanoporous palladium in the electrochemical double-layer regime prior (black) and after cyclic hydrogen absorption following the charging sequence in Figure 5 (green). Voltammograms were recorded at a scan rate of 0.5 mV·s^−1^ in 1 M KOH.

In order to evaluate structural coarsening, which is associated with a reduction in active surface area, cyclic voltammograms in the electrochemical double layer regime were recorded on an equivalent npPd sample before and after the thirteen-step absorption procedure, shown in Figure 5. Results are depicted in Figure 6. The CV after cyclic absorption (green curve) shows a reduction in double layer current of about 25% compared to the pristine, untreated npPd sample (black curve). The black curve shows a stronger contribution of pseudocapacitive surface charging, indicated by the larger deviation from the rectangular shape of ideal capacitors. A comparison of double-layer currents allows one to evaluate changes in the specific surface area of npPd upon hydrogenation (see section Discussion).

### Galvanostatic hydrogen desorption

**Figure 7:**
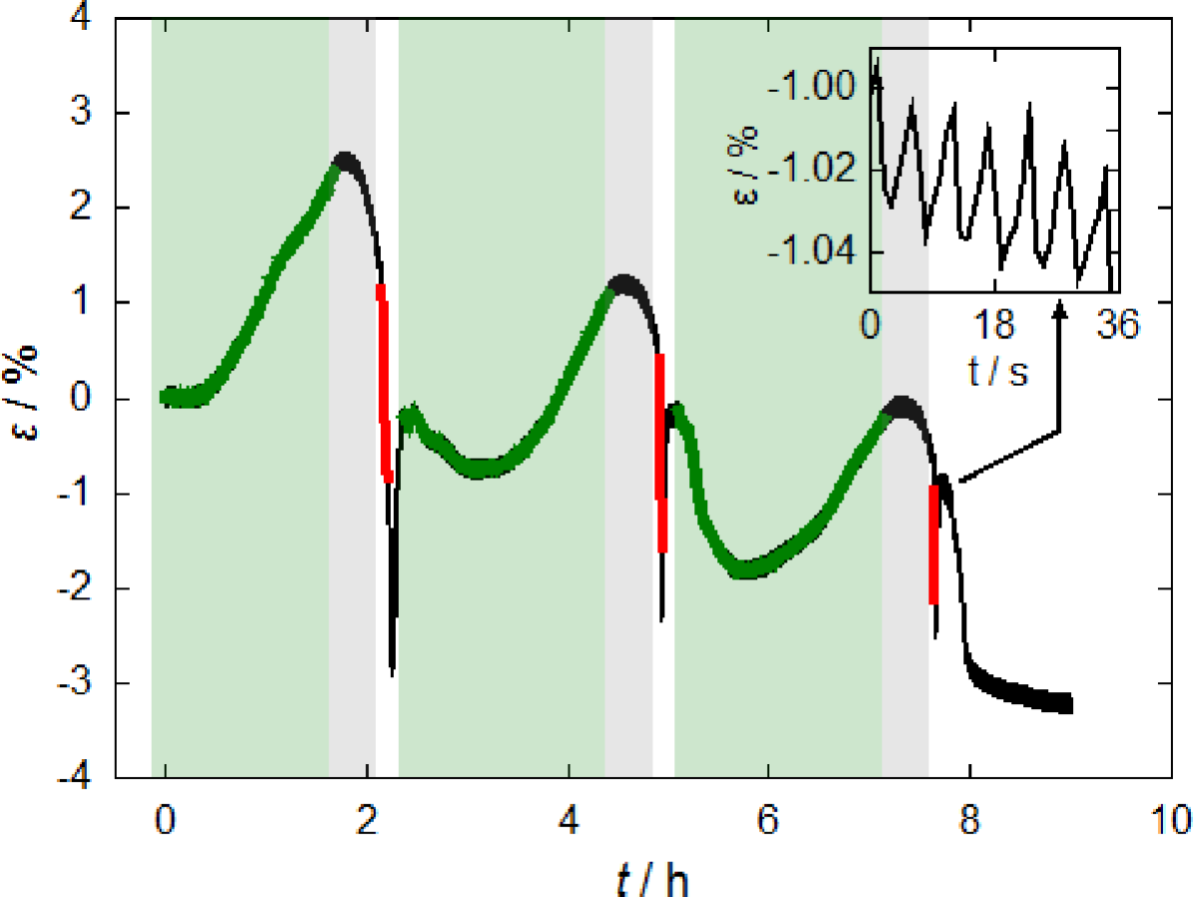
Strain ε as a function of the time *t* for constant-current/constant-potential (2 mA – green, −1 V – grey bars) loading of hydrogen in nanoporous palladium followed by galvanostatic unloading at 32 mA, 64 mA and 128 mA (red) in 1 M KOH solution. The inset shows an enlarged section of the curve after constant current desorption at 128 mA.

To investigate a possible dependence of the strain on the unloading rate, galvanostatic hydrogen-desorption experiments were performed, which are shown in Figure 7. For this purpose, a npPd sample was charged with hydrogen to a fixed concentration in the PdH_β_-phase and discharged at a fixed current, which in a first approximation should be proportional to the strain rate upon discharging. To achieve a defined loading state, a constant-current (green)/constant-potential (grey bars) charging procedure, inspired by a typical battery charging process, was implemented. As soon as the potential monitored during the constant-current (2 mA) loading crossed −1 V, the potential was held constant at this value until the current dropped below 0.5 mA. For the discharging experiments three different currents of 32 mA, 64 mA, and 128 mA were applied. After each unloading step, a wait time of 5 min without external potential was set to allow for sample equilibration and desorption of residual hydrogen.

The strain in Figure 7 shows a pronounced increase upon constant-current loading (green) followed by a small decrease during the constant-potential loading procedure (grey bars). Upon galvanostatic unloading (red) the strain drops almost linearly. Evaluation shows that the slope of these lines is approximately proportional to the unloading current. The black part of the curve directly after discharging, corresponds to the wait time, allowing the sample to relax. Once the strain passed the overshoot and reached a stable value, serrations (as shown in the inset) could be observed in the strain curve. These fluctuations were significantly larger than thermal fluctuations in the strain curves.

## Discussion

In the following, the particular strain responses of npPd upon electrochemical hydrogen desorption, namely the strain overshoot, the strain offset and the high-current strain serrations, will be illuminated. The initial part of the discussion addresses the palladium-hydride phase transition, as the concept is of utmost importance for the mechanical behaviour of npPd.

### Current understanding of palladium-hydride phase transitions

The nature of the phase transition in palladium hydride crucially depends on both sample dimensions and the rate of hydrogen uptake and removal. During hydrogen loading the PdH_α_-to-PdH_β_ transition was reported to proceed coherently in palladium nanoparticles, while the reverse transition can be either coherent or incoherent, depending on the particle size [34–35]. In bulk palladium the transition from PdH_β_ to PdH_α_ is known to proceed incoherently, inducing dislocations to reduce internal stresses [34]. A coherent phase transition on the other hand involves the occurrence of internal stresses due to spatial variations of the lattice spacing, while no dislocations are induced. Therefore, particles must be sufficiently small in order to maintain surface–shell–core coherency during the α-to-β transition [34]. A study on phase transitions in PdH thin films concluded that coherent transitions are possible up to a critical film thickness of 22 nm [36]. As ligament sizes in npPd produced by dealloying in our work are in the order of 20 nm, palladium hydride phase transitions can be expected to proceed mainly coherently as in nanoparticles up to a size of 45 nm [34], although ligament interconnections in the nanoporous network could enlarge the local structure size.

At low sorption rates it has been shown that upon hydrogen desorption the PdH_β_-to-PdH_α_ transition follows a classical nucleation-and-growth mechanism, i.e., the α-phase forms nuclei in the β-matrix that begin to grow [37]. During the hydride phase transition lattice constants of both phases may differ from their equilibrium values, which are quoted in the introduction. In situ X-ray diffraction experiments pointed towards a reduced lattice constant for PdH_α_ [38] when it appeared again during desorption. In the course of the desorption-induced phase transition the lattice constant of the α-phase increases with the growing amount of PdH_α_ (lattice-constant relaxation). When the PdH_β_-phase vanished, the lattice constant of PdH_α_ was reported to be higher than its value before initial absorption, indicating an irreversible effect of the hydrogen treatment on the lattice [38]. It is important to note that also during absorption a decrease in lattice constant for PdH_α_ was observed as soon as PdH_β_ started to form. The reduced lattice constant of the α-phase, which will be referred to as α-phase “straining” in the following, is related to internal stresses arising during the phase transition [37].

The driving force for the reduction of the lattice constant remains to be clarified, but an elastic compression of the α-nuclei due to a structure-induced compressive stress at the solid–electrolyte interface is a plausible mechanism. As the PdH_α_-phase nucleates in a PdH_β_-matrix, an additional compressive stress might be present due to the expanded lattice of the β-matrix, as already suggested in [38]. Both stresses, i.e., from the structure and from the β-matrix, could confine the α-phase elastically to a smaller lattice constant.

When precipitates grow in the course of hydrogen desorption at a certain critical size a transition from coherent-to-incoherent (or coherent-to-semicoherent) is energetically favoured, which has been studied experimentally via small-angle neutron scattering [39]. The transition is expected not only to relief the stresses at the coherent α–β interfaces, but also the internal compressive stresses found in the α-phase, leading to an increase in the lattice parameter of the α-phase.

### α-phase straining – strain overshoot

The strain overshoot visible in Figure 3, Figure 5 and Figure 7 can be explained by the aforementioned straining of the α-phase (see previous section) as discussed in the following. The overshooting effect is most prominent during the desorption process in Figure 3. The monotonous length decrease in region (c) in Figure 3 can be attributed to hydrogen desorption from the PdH_β_-phase and a concomitant formation of strained PdH_α_-nuclei. Region (d), where the strain starts to increase again, can be assigned to the process of lattice-constant relaxation in the α-phase, while the fraction of PdH_β_ is reduced. Region (e) corresponds to the desorption from PdH_α_, which is the predominant phase at this point, until the sample is completely dehydrogenated.

The same strain overshoot was monitored during desorption after galvanostatic loading and can be followed evolving over time in Figure 5. Considering a single absorption/desorption experiment as in the inset in Figure 5 one can easily recognise regions (c), (d) and (e) described above for Figure 3 in the red part.

In contrast to Figure 3, the straining of the α-phase can be observed directly in the experiments upon absorption (black curves, Figure 5): Between cycles 5 and 11 the strain curves in black show a falling tendency after an initial increase. As the straining of the α-phase is connected with internal stresses, it can only be observed in the region of phase coexistence of PdH_α_ and PdH_β_. As soon as the voltage of PdH_α_ depletion (−0.99 V) is crossed in the 11th cycle of the galvanostatic loading series, a continuous length increase upon absorption due to expansion of the β-phase is observed in Figure 5, as a straining of PdH_α_ is obviously no longer a possible mechanism.

In Figure 7 the strain overshoot can be monitored in a different form. Straining of the α-phase occurs mainly in the region of constant-potential loading (grey bars in Figure 7), observable as decrease in the strain curve. Once the galvanostatic desorption starts (red) the sample contracts as expected with a strain rate approximately proportional to the unloading current. The relaxation of the strained α-phase appears without an externally applied potential or current (black after unloading in red), resulting in a similar overshooting effect.

As indicated above, a possible straining of the PdH_α_-regions in our experiments might be connected with the high surface stress exerted by the nanoporous structure, which could also account for the drastic changes in length even below the equilibrium value at the start of absorption (e.g., inset Figure 5, black curve). In this context it is worth mentioning a recent study on hierarchical npPd [20], which pointed towards a positive (tensile) surface stress in the PdH_β_-phase, thus a bulk contraction, and a negative (compressive) surface stress in the PdH_α_-phase, thus a bulk expansion. This is in agreement with the straining of the PdH_α_-phase in the PdH_β_-matrix, as a bulk compression by the PdH_β_-phase can also affect the PdH_α_-nuclei, provided they have the lower volume fraction. Nucleation of the PdH_α_ on the sample surface attenuates the compressive effect by PdH_β_, compared to nuclei surrounded by the β-phase. Overshooting behaviour is still plausible in this case, although with a reduced strain amplitude. Lattice reorientations driven by surface stress, suggested by simulations in both pristine [40] and hydrided Pd nanowires [41], could also be indicative for the concept of a surface-stress contribution to the α-phase straining.

Finally, it should be noted that the stresses responsible for the overshooting behaviour must be different in nature from misfit stresses at a coherent phase boundary. As a α-phase coherently matched to the β-phase would experience a tensile straining of the lattice constant, the above described compressive straining cannot be a result of these coherent interfaces.

### Internal-stress plasticity – strain offset

**Figure 8:**
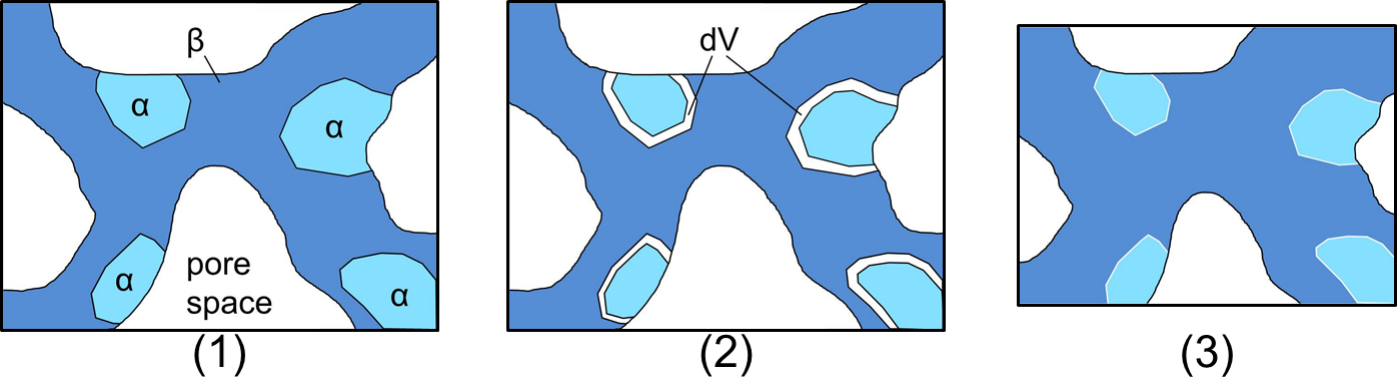
Schematic representation of the plasticity evolution during hydrogen desorption from nanoporous palladium hydride. In a saturated PdH_β_-phase PdH_α_-nuclei start to form (1) in the course of the desorption process. The strain mismatch (dV) of the two phases emerging during the phase transition (2) is then biased by the surface tension and leads to plastic deformation of the nanoporous structure (3) in radial direction, so that surface area is reduced. Note that the total palladium volume remains unchanged during this transformation.

The irreversible contractions after each absorption/desorption cycle observed in Figure 3 (−0.5%) and Figure 5 (e.g., cycle 13, −6.5%) are a result of the internal-stress plasticity mechanism that is elaborated below. The most important factor related to the irreversible contraction upon desorption after hydrogenation at low potentials are the forces which the sample is subjected to during dilatometry. On the one hand the sample experiences a weak, but steady compressive stress by means of the dilatometer pushrod. On the other hand, an additional driving force for compressive deformation due to the nanoporous structure itself and its surface excess energy is present in the sample. An effect called internal-stress plasticity (ISP), also commonly known as transformation plasticity, is well established in the literature [42–45]. A schematic representation of the transformation-mismatch plasticity mechanism in npPd is presented in Figure 8. A driving force for deformation, which is present from the two different sources in our case, biases internal strains emerging in the course of a phase transition in a preferred (radial) direction. This leads to an effective yielding in that direction [42]. We suppose that the transition from coherent to incoherent (or semicoherent) precipitates, triggers the ISP mechanism in our case. Both internal strains and a driving force for compression are necessary to activate this mechanism of plastic deformation.

The phase transition that induces the required mismatch strains for the plasticity enhancement is again the PdH_β_-to-PdH_α_ transition. In combination with the driving force being the reduction in surface energy, ISP can be observed during hydrogen desorption in nanoporous samples. The high plastic-deformation strains up to 6.5% during one single phase-transition cycle in Figure 5 are a result of this mechanism. As the plastic deformation during a single absorption/desorption cycle is, in a first-order approximation, proportional to the external stress [42,45], the strong deformation can be related to the surface-stress-induced compression in nanoporous samples, which is considerably higher than that in bulk samples. Hydrogen-induced phase transitions leading to ISP were reported for titanium [44], zirconium [46] and palladium [42] before. For a palladium wire an extraordinarily large deformation of ≈40% was reached under tensile load upon repetitive hydrogen loading and unloading in a long-term experiment [42]. In principle, such deformations should also be attainable for npPd by enlarging the number of absorption/desorption cycles.

Recent works on plasticity in nanoporous gold have pointed towards the surface tension (in units of energy per area) being the relevant capillary parameter for plastic deformation, rather than surface stress, which is only responsible for elastic contributions [47–48]. This is reflected in an asymmetric deformation behaviour in compression and tension as shown by Lührs et al. [48], where expansion under a tensile load is inhibited and a contraction under compressive force is promoted. Mameka et al. reported that surface-tension-driven deformation leads to changes in the total surface area, as supported by lower surface areas measured after compression tests [47]. Therefore, in our case of nanoporous palladium under compression a coarsening of the structure can be expected, giving rise to the length contraction. It has been shown using molecular dynamics (MD) simulations that plastic deformation at sufficiently high strains in general does promote structural coarsening in nanoporous materials [49]. The predicted coarsening of npPd upon (hydrogen-induced) deformation in our case is supported by CV measurements in the double-layer region (Figure 6). Double-layer currents from cyclic voltammograms are commonly used to calculate the electrochemical double-layer capacitance *C*_d_ and the related real surface area *A*, following the relation:

[2] $$A=\frac{C_{\text{d}}}{C_{\text{s}}}=\frac{I_{\text{d}}}{sC_{\text{s}}}\;,$$

where *C*_s_ denotes the specific capacitance per unit area, *I*_d_ the double-layer current and *s* the scan rate at which the CV was recorded. As the capacitance per unit area *C*_s_ is a constant value for an electrode surface in a certain electrolyte, this capacitance values are directly proportional to real surface areas [50]. To date, no such reference values exist for palladium in potassium hydroxide solution. Nonetheless, the observed reduction of the double-layer current by about 25% in Figure 6 must lead to a surface area reduction of the same percentage. As the ligament diameter is related to the reciprocal specific suface area (SSA) in disorderd nanoporous structures [51], a decrease of SSA would give rise to an increase in ligament size. Assuming a constant relative density, one can give an upper boundary for the ligament-size increase of about 33% [51]. The initial ligament size of approximately 20 nm should accordingly coarsen to ligament sizes not larger than 27 nm. This increase in ligament size, derived from double layer current variations, is a strong indication that the ISP deformation mechanism is active in npPd.

Plastic deformation up to 6.5% after a single hydrogen-loading/unloading cycle in nanoporous palladium samples prepared in this work (see Figure 5) is seemingly in striking contrast with the remarkable hydrogen-cycling stability over 1000 cycles reported for hierarchical nanoporous palladium by Shi et al. [20]. However, closer inspection suggests that the mechanism proposed here leading to plastic deformation might not be available in hierarchical npPd prepared in the mentioned work. The smaller structural size of the finer ligament structure below 10 nm in the work of Shi et al. might allow for a fully coherent phase transition completely avoiding the creation of incoherent phase boundaries and thus defects also upon desorption, which is an idea already brought up by the authors of the mentioned publication. In the case of npPd produced in this work phase coherency is only partly possible upon hydrogen desorption for our nanoporous samples indicated, e.g., by serrations in the strain (see next section). As the transition from coherent-to-incoherent (or coherent-to-semicoherent) interfaces of PdH_α_ and PdH_α_ phases is supposed to initiate deformation, this could explain the discrepancies between the mechanical performance of the two variants of npPd. In addition the contribution of dislocation plasticity is avoided when the phase transition proceeds fully coherently.

Crossing the threshold potential of PdH_β_-phase formation (−0.96 V) in our electrochemical experiments enables plastic deformation following the ISP mechanism, which is the foundation of plasticity control in npPd. Only if the β-phase is formed upon hydrogenation (starting in cycle 5 in Figure 5) plasticity occurs during the desorption process. Theoretical aspects of the strain response, including both surface stress and plasticity mechanism, are discussed in the Appendix section.

### Dislocation plasticity – strain serrations

The strain serrations during high-current desorption processes, visible in the inset in Figure 7, indicates the presence of an additional deformation mechanism of npPd based on dislocation activity, which is subject of this section.

Two different mechanisms of plasticity evolution are common in hydrogen-treated metals: hydrogen embrittlement [52–53] and hydrogen-enhanced localised plasticity [54]. In a simplistic view, hydrogen embrittlement evolves as a result of dislocations introduced by interstitial hydrogen atoms, which remain in the crystal lattice and hinder dislocation movement, even after complete hydrogen desorption in an ideal lattice. Hydrogen-enhanced localised plasticity, on the other hand, is a result of stress-field shielding by solute hydrogen atoms and thus an enhancement of dislocation mobility. The appearance of these converse phenomena strongly depends on the grain size. In small grains hydrogen-enhanced localised plasticity is dominant, while in coarse grains hydrogen embrittlement is the prevalent mechanism [55]. Hydrogen–metal interactions are still not fully understood and demand further research.

The ISP mechanism, responsible for the irreversible contraction (strain offset, see previous section), is based on the coherency of phases during the transition, which does not introduce dislocations in the nanoporous structure. However, a fully coherent transition can be hampered at high sorption and desorption rates, which may give rise to additional dislocation plasticity.

The strong fluctuations observed in the inset in Figure 7 point towards a dislocation plasticity contribution. Serrations are common features in stress–strain curves as a result of dynamic strain aging (DSA) [56]. DSA is related to dislocation interactions with obstacles in the lattice, which may be other lattice defects or solute atoms. The effect is activated at high strain (and thus desorption) rates, where solutes fail to keep up with the rapidly moving dislocations [56]. Hydrogen solute atoms were reported to cause DSA in α-Ti [56] at low concentrations. The Portevin–Le Chatelier effect, which is directly related to the DSA mechanism, was also reported for hydrogenated palladium [57]. Arrested dislocations might also be possible in npPd. Hydrogen is present as solute atom in the PdH_α_-phase, which is the predominant phase after (incomplete) galvanostatic desorption. Arrested dislocation movement, however, cannot solely account for the sawtooth-shape of the strain curve in the inset in Figure 7. Since dislocation arrest simply corresponds to a constant sample length, the strain curve would decrease in a step-like manner, but no length increase would be observed as it is the case in Figure 7. Nonetheless, a combination of dislocation arrest, being responsible for the descending part of the sawtooth, with the PdH_α_-phase relaxation introduced above, accounting for the ascending part of the sawtooth, could be a possible explanation for the serrations in the strain curve.

## Summary

In this work we investigated the deformation mechanisms in npPd by using an in situ dilatometric technique in an electrochemical environment. Different hydrogen-sorption experiments, controlled by either current or potential, were used to induce phase transitions in npPd in a controlled manner and to evaluate the influence of PdH_α_- and PdH_β_-phase on the strain response. Plasticity mechanisms based on both internal stresses and dislocation activity were found to be active in nanoporous palladium hydrides, leading to a peculiar strain curve. A phase-transition upon dehydrating in combination with the extraordinarily high surface stress, due to the nanoporous structure, is responsible for this uncommon strain response. A maximum compressive plastic deformation of ≈6.5% could be attained during a single hydrogen sorption–desorption-cycle. A phenomenological description of plastic deformation in npPd led to the following conclusions:

- The driving force of plastic deformation in nanoporous palladium is the surface stress.
- A coherent hydrogen-induced phase transition from PdH_β_ to PdH_α_ during hydrogen desorption enables the mechanism of internal-stress plasticity in nanoporous palladium.
- This phase transition, and thus plasticity, can be controlled via the potential in an electrochemical environment. A threshold potential for nanoporous PdH_β_ formation (−0.96 V vs Ag/AgCl in 1 M KOH) was determined.

## Experimental

### Alloy fabrication

A Co_75_Pd_25_ master alloy was prepared from Pd granules (AlfaAesar, 99.95%) and Co slug (AlfaAesar, 99.95%) via electron-beam melting. The sample was melted multiple times to ensure complete intermixing in the liquid state and consequently a homogeneous single-phase alloy. The produced alloy drop was thinned to a platelet (4–5 mm in height) using a screw press. Homogeneity of the alloy was confirmed by XRD-measurements. Further processing consisted of several consecutive rolling and annealing steps until a thickness of 270 μm was reached. The annealing steps were conducted in a vacuum furnace at 700 °C and 10^−5^ mbar for 1 h. The resulting foil was cut into squares of 5 × 5 mm^2^.

### Dealloying

The setup used for in situ dilatometry during electrochemical characterisation was similar to that described in an earlier work of our group [17]. For electrochemical dealloying the square-shaped samples were placed in a Linseis L75 vertical pushrod dilatometer, operating at a constant force of 100 mN. Electrical contact to an Autolab PGSTAT204 potentiostat was established using an annealed Pd wire (ChemPur, 99.95%). A coiled Pd wire and an Ag/AgCl (sat. KCl) electrode (Metrohm) served as counter and reference electrode, respectively. Electrochemical dealloying was conducted in 0.1 M sulfuric acid solution at a potential of +0.55 V (vs Ag/AgCl), a method that is commonly used to achieve homogeneous nanoporous palladium structures [16–17]. Dealloying was stopped at currents below 0.1 mA. The residual cobalt concentration in nanoporous palladium prepared via this route was reported to be below 2 atom % [58].

### Electrochemical cell setup

After dealloying, the samples were rinsed in distilled water for several minutes, before immersing them in 1 M KOH aqueous solution. For electrochemical characterisation a porous carbon cloth was used as counter electrode. In order to assure measurement stability a pre-treatment consisting of five voltammetric cycles was applied at a scan rate of 0.1 mV·s^−1^ in a potential window between −1 V and 0.4 V. All potentials in the text below refer to the Ag/AgCl (sat. KCl) reference electrode. Zero on the strain axis in the plots was chosen in the electrochemical double-layer regime. For the distinction of hydride phases in npPd, samples were dealloyed and characterised ex situ in a standard three-electrode electrochemical cell, using the same counter and reference electrodes as described for the in situ setup.

As chemical dissolution processes can hardly be discerned from mechanical yielding on the basis of strain curves obtained in a dilatometer, dissolution processes should be minimised using an adequate electrolyte. Aqueous potassium hydroxide solution enables measurement stability in a broad potential window [17–18], while mostly surpressing chemical dissolution of palladium and palladium oxide [29], making it the preferred electrolyte for studies in this work.

## Appendix

### Theoretical considerations

The theoretical basis to evaluate the experimental strain response of npPd upon hydrogen absorption and desorption will be summarised in the following. As indicated above, the irreversible, plastic length changes of nanoporous palladium measured in the dilatometer are results of two different stresses: the stress resulting from the external force applied by the dilatometer (σ_dil_) and the axial component of the surface stress of the nanoporous structure (σ_ss_). σ_dil_ is of the order of 10 kPa for dilatometric experiments, while σ_ss_ is related to the ligament size reciprocally:

[3] $$\sigma_{\text{SS}}=\frac{3.7f}{D}\;.$$

The factor 3.7 is a dimensionless constant calculated for arbitrary porous structures [51], *f* is the surface stress (for a Pd surface: *f* ≈ 1.9 N/m [59]) and *D* the ligament size. With ligament sizes of the order of 20 nm, an axial component of the surface stress of about 350 MPa would be obtained. Considering this high value one can exclude a significant contribution of the dilatometric stress to the deformation in nanoporous palladium. Both stresses contribute to elastic and plastic deformation of the sample. An expression for the elastic component of the length changes in a nanoporous material, which has been introduced for nanoporous gold recently [60], is

[4] $$\Delta \varepsilon_{\text{e}}=-\frac{2\alpha }{9K}\Delta f-\frac{\sigma_{\text{dil}}}{E_{\text{np}}}\;,$$

where Δε_e_ denotes the change in elastic strain, α is the specific surface area *A*/*V*, *K* the bulk modulus, Δ*f* the change in surface stress, and *E*_np_ the Young’s modulus of the nanoporous structure. Δ*f* can be linked to the change in surface charge density Δ*q* via electrocapillary coupling coefficients ζ in different electrochemical regimes. Similar relationships have been successfully utilised to bias dilatometric elastic-strain responses by means of electrochemical surface charging in nanoporous systems [3,61–63].

Note that the absolute value of the surface stress (*f*) does not contribute to any elastic length changes, but variations of this quantity do (Δ*f*). The plastic component of the total strain, which is a result of the ISP mechanism, is described by the theoretical model of Greenwood and Johnson [45] for sufficiently low stresses compared to the yield strength. In the present work we propose a slightly altered version of this relationship, linking plastic deformation to surface stress:

[5] $$\Delta \varepsilon_{\text{p}}=\frac{5}{3}\frac{\Delta V}{V}\frac{\left(\sigma_{\text{dil}}+\sigma_{\text{ss}}\right)}{\sigma_{\text{y}}}\;,$$

where Δε_p_ is the plastic strain per phase-transformation cycle, Δ*V*/*V* the volume mismatch of the two involved phases (for Pd and PdH_β_: Δ*V*/*V* ≈ 0.1), and σ_y_ the yield strength of the nanoporous palladium. As pointed out in the discussion the actual driving force for plastic deformation is surface tension. From surface tension, which is a scalar quantity, it is not possible to determine a corresponding surface stress, which is a tensor quantity, without additional information. The surface stress σ_ss_ in Equation 5 therefore refers to a virtual stress, representing the effect of surface tension on plastic deformation.

The yield strength of npPd also strongly depends on the ligament size, following a relation similar to the Hall–Petch equation [64]:

[6] $$\sigma_{\text{y}}=\sigma_{\text{0}}+kL^{-0.5}\;.$$

σ_0_ denotes the yield strength of bulk palladium and *k* is a material-specific constant, yet to be determined for nanoporous palladium. It has been shown for npAu, which follows a similar scaling equation for both yield strength and surface stress, that σ_SS_ is well below σ_y_ for ligament sizes down to 10 nm [24]. This allows to use Equation 5, which is only valid in the regime of low stresses. At larger stresses the plastic strain is connected with the applied stress via a more complex, nonlinear relationship [43]. The plastic strain component is, in contrast to the elastic deformation, proportional to the absolute value of the surface stress (*f*) in our proposed equation (Equation 5). Adding a term accounting for the work-hardening process during a phase transition should be considered for a more accurate description of the property evolution.

Many properties in the above-mentioned equations can not be assumed to be constant during our experiments. Besides the potential-dependence of the surface stress *f*, which is utilised to trigger the strain response, it is well known that *E*_np_, *D*, α and σ_np_ are all potential-dependent properties, which makes the above-stated equations hard to evaluate during measurements in practice. The given framework of equations might serve as a starting point for a detailed theoretical treatment of the mechanical properties of npPd in the future.
